# Sulfur assimilation using gaseous carbonyl sulfide by the soil fungus *Trichoderma harzianum*

**DOI:** 10.1128/aem.02015-23

**Published:** 2024-02-01

**Authors:** Ryuka Iizuka, Shohei Hattori, Yusuke Kosaka, Yoshihito Masaki, Yusuke Kawano, Iwao Ohtsu, David Hibbett, Yoko Katayama, Makoto Yoshida

**Affiliations:** 1 Graduate School of Agriculture, Tokyo University of Agriculture and Technology, Fuchu, Tokyo, Japan; 2 International Center for Isotope Effects Research, School of Earth Sciences and Engineering, Nanjing University, Nanjing, Jiangsu, China; 3 Faculty of Life and Environmental Sciences, University of Tsukuba, Tsukuba, Ibaraki, Japan; 4 Euglena Co., Ltd., Minato‑ku, Tokyo, Japan; 5 Department of Biology, Clark University, Worcester, Massachusetts, USA; 6 Independent Administrative Institution, Tokyo National Research Institute for Cultural Properties, Taito-ku, Tokyo, Japan; Chalmers tekniska hogskola AB, Sweden

**Keywords:** fungi, sulfur metabolism, fixation, carbonyl sulfide, evolution

## Abstract

Fungi have the capacity to assimilate a diverse range of both inorganic and organic sulfur compounds. It has been recognized that all sulfur sources taken up by fungi are in soluble forms. In this study, we present evidence that fungi can utilize gaseous carbonyl sulfide (COS) for the assimilation of a sulfur compound. We found that the filamentous fungus *Trichoderma harzianum* strain THIF08, which has constitutively high COS-degrading activity, was able to grow with COS as the sole sulfur source. Cultivation with ^34^S-labeled COS revealed that sulfur atom from COS was incorporated into intracellular metabolites such as glutathione and ergothioneine. COS degradation by strain THIF08, in which as much of the moisture derived from the agar medium as possible was removed, indicated that gaseous COS was taken up directly into the cell. *Escherichia coli* transformed with a COS hydrolase (COSase) gene, which is clade D of the β-class carbonic anhydrase subfamily enzyme with high specificity for COS but low activity for CO_2_ hydration, showed that the COSase is involved in COS assimilation. Comparison of sulfur metabolites of strain THIF08 revealed a higher relative abundance of reduced sulfur compounds under the COS-supplemented condition than the sulfate-supplemented condition, suggesting that sulfur assimilation is more energetically efficient with COS than with sulfate because there is no redox change of sulfur. Phylogenetic analysis of the genes encoding COSase, which are distributed in a wide range of fungal taxa, suggests that the common ancestor of Ascomycota, Basidiomycota, and Mucoromycota acquired COSase at about 790–670 Ma.

**IMPORTANCE:**

The biological assimilation of gaseous CO_2_ and N_2_ involves essential processes known as carbon fixation and nitrogen fixation, respectively. In this study, we found that the fungus *Trichoderma harzianum* strain THIF08 can grow with gaseous carbonyl sulfide (COS), the most abundant and ubiquitous gaseous sulfur compound, as a sulfur source. When the fungus grew in these conditions, COS was assimilated into sulfur metabolites, and the key enzyme of this assimilation process is COS hydrolase (COSase), which specifically degrades COS. Moreover, the pathway was more energy efficient than the typical sulfate assimilation pathway. COSase genes are widely distributed in Ascomycota, Basidiomycota, and Mucoromycota and also occur in some Chytridiomycota, indicating that COS assimilation is widespread in fungi. Phylogenetic analysis of these genes revealed that the acquisition of COSase in filamentous fungi was estimated to have occurred at about 790–670 Ma, around the time that filamentous fungi transitioned to a terrestrial environment.

## INTRODUCTION

Sulfur is an essential element for all organisms, and sulfur compounds are responsible for important physiological functions, such as the synthesis of sulfur-containing amino acids and cofactors (1, 2), redox signaling (3), and energy metabolism (4). Fungi are known to assimilate sulfur in the form of organic molecules, such as methionine and homocysteine, as well as inorganic molecules, such as sulfate and thiosulfate (5–9). Gaseous sulfur compounds are ubiquitous trace constituents in the atmosphere, but direct uptake and assimilation of gaseous sulfur compounds by fungi and other organisms as a sulfur source have not been reported.

The most abundant gaseous sulfur compound in the atmosphere is carbonyl sulfide (COS or carbon oxysulfide), existing at a concentration of around 500 pptv in the troposphere (10, 11). In contrast to other gaseous sulfur molecules, such as dimethyl sulfide, sulfur dioxide, and hydrogen sulfide (H_2_S), which are oxidized to sulfate in the troposphere within 1.0 day, 1.2 days, and a few hours, respectively (12, 13), COS is stable, with a lifetime of ~4.3 years (14). Atmospheric COS is degraded biologically by plants and microorganisms. In plant leaves, gaseous COS is degraded by the side reaction of enzymes that interact with CO_2_, such as RuBisCO or carbonic anhydrases (CA) during photosynthesis (15–17), although the physiological role of this reaction is unknown. In addition, a variety of bacteria and fungi can degrade COS (18–21); however, their physiological role is still unknown, except in relation to the energy metabolism of chemolithoautotrophic sulfur oxidation: *Thiobacillus thioparus* (*T. thioparus* hereafter) strain THI115 possesses COS hydrolase (COSase, COS + H_2_O → CO_2_ + H_2_S), which is clade D of the β-class CA (β-D-CA) subfamily enzyme with high specificity for COS but low activity for CO_2_ hydration, and COS is degraded by COSase into H_2_S and further converted into sulfate to be an end product of energy production in the chemolithotrophic metabolism system (18, 22). On the other hand, COS-degrading activity involving β-D-CA (19–21) has been reported in many heterotrophic bacteria and fungi, but the physiological role of COS degradation is unknown.

Several fungi isolated from soil can degrade COS at atmospheric concentration levels (500 pptv) even to extremely high concentration levels exceeding 10,000 ppmv, which is also the case of bacteria (23, 24). Ecological studies have shown that in soil, which is one of the major sinks for atmospheric COS (25–27), fungi are the important degraders of COS, and that the key enzyme for soil COS degradation was determined as β-D-CA (28), which are distinct from plant β-CAs involved in CO_2_ assimilation (29, 30). We have experimentally identified β-D-CA to be a COSase in the filamentous fungus *Trichoderma harzianum* (*T. harzianum* hereafter) strain THIF08, which was isolated from forest soil (24, 31). These findings suggest that COS degradation, which is a side reaction in plants, is catalyzed by an enzyme that plays a specific role in fungi. However, the physiological role of fungal COSase remains unclear, as the biological fate of the sulfur atoms in the COS degradation products is unknown.

Based on the knowledge described above, we hypothesized that fungi take up gaseous COS via COSase for sulfur assimilation. In this study, using *T. harzianum* strain THIF08 (NBRC 115644), we investigated the effect of COS on mycelial growth, the behavior of sulfur metabolites using COS as a sulfur source by metabolomic analysis, and the function of COSase using a recombinant *E. coli*. Furthermore, based on the phylogenetic analysis, we discuss the molecular evolution of COSase.

## RESULTS AND DISCUSSION

### Fungal growth with COS as the sole sulfur source

**Fig 1:**
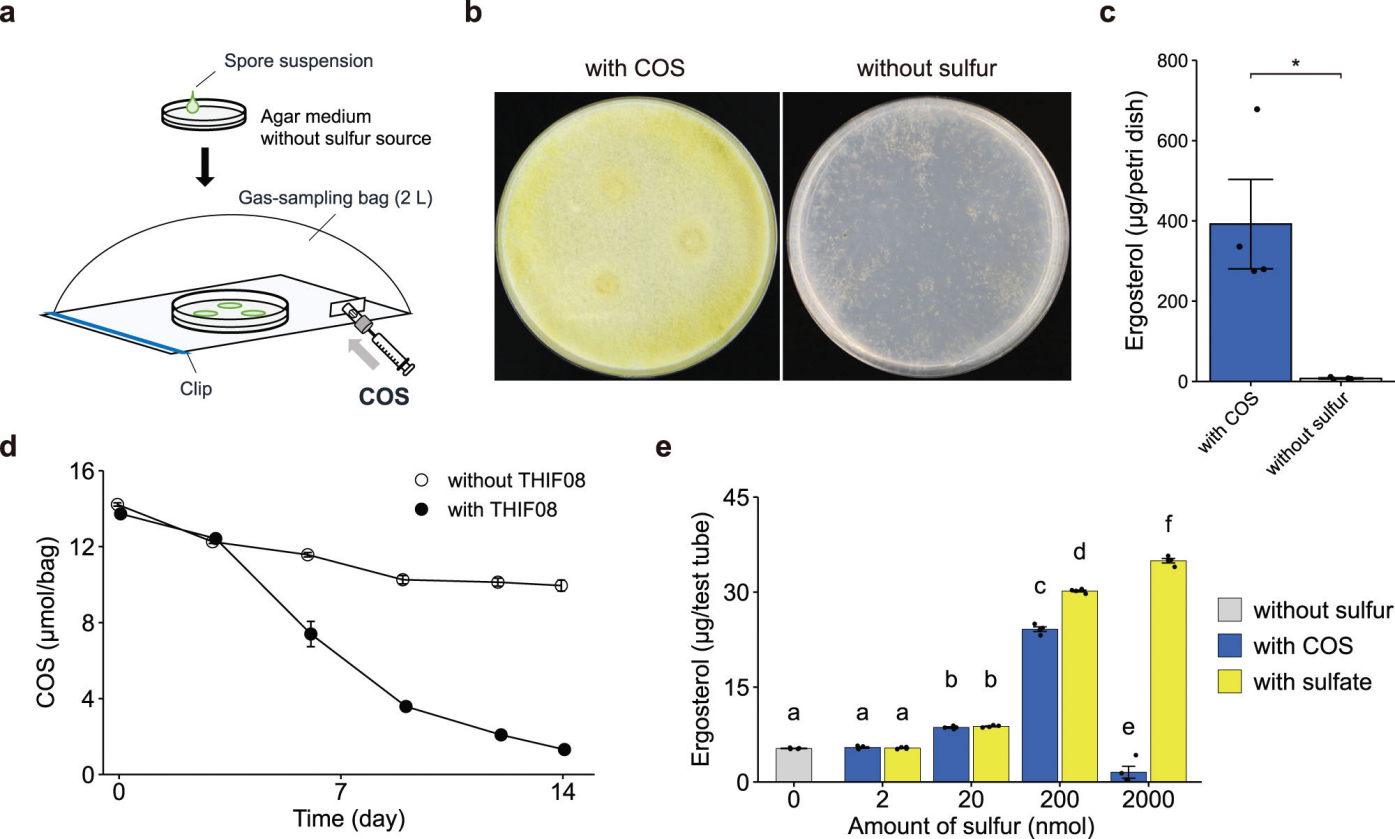
Growth of strain THIF08 with COS as the sole source of sulfur. (a–d) *T. harzianum* strain THIF08 was cultivated on a synthetic agar medium with no sulfur source added and instead COS was added as the sole sulfur source in the 2 L headspace of the sampling bag for 2 weeks. (a) Schematic diagram of the experiment of cultivation using the sampling bag. (b) Comparison of the proliferation of mycelium on the agar plate. Left, with 16 µmol COS in 2 L headspace. Right, without COS. (c) Comparison of biomass expressed by the amounts of ergosterol between cells cultured in the presence of COS as a sole sulfur source or without sulfur source (*n* = 4). *P < 0.05. (d) Time course changes of the amount of COS in culture systems inoculated by strain THIF08. Filled circles: inoculated strain THIF08; open circles: no inoculation (*n* = 4). (e) Growth of strain THIF08 with 2, 20, 200, or 2,000 nmol COS or sulfate as the sole sulfur source in the test tube after 8 days of incubation (*n* = 4). Statistical significance was determined using a one-way ANOVA followed by Tukey’s *post hoc* test to identify specific group differences, with a significance level set at *P* < 0.05. In panels c–e, data are means ± s.e.m. *n* represents independent biological replicates.

*T. harzianum* strain THIF08 was cultivated on agar medium in a Petri dish with and without gaseous COS as the sole source of sulfur in a sampling bag (2 L headspace, 5 L Tedlar Bag; GL Sciences) (Fig. 1a). Vigorous growth of the yellowish cells over the entire area was observed only under COS-supplemented conditions (Fig. 1b). The amount of ergosterol (a component of fungal cell membranes and an indicator of growth) after 2 weeks of incubation was more than 50 times higher in cultures with COS than without COS (Fig. 1c). Furthermore, the amount of COS in the headspace decreased during cultivation (Fig. 1d). The COS was slightly decreased under the condition without strain THIF08, which is expected to be due to hydrolysis by water in the medium, but COS degradation was more clearly observed in the condition with strain THIF08. These results indicate that strain THIF08 can utilize gaseous COS as the sole source of sulfur.

To test the effect of different sulfur sources on the growth of strain THIF08, fungal growth was compared in slant cultures, with either COS in the 22 mL headspace or sulfate in the agar medium (Fig. 1e). The growth of strain THIF08 under COS-supplemented conditions increased gradually with the addition of COS and was similar to sulfate cultures at 20 nmol of sulfur and significantly increased with 200 nmol, though slightly less than in sulfate cultures (Fig. 1e). On the other hand, at 2,000 nmol COS-supplemented condition in 22 mL headspace (which corresponds to 2,200 ppmv), the growth of strain THIF08 was even lower than in cultures without any sulfur source. This reduction might be due to the inhibition of spore germination and mycelial growth by H_2_S (32), which is a COS degradation product. Indeed, when 200 nmol H_2_S was added as the sole sulfur source, the growth of strain THIF08 was inferior compared to that with the same amount of COS added (Fig. S1). The slight increase in growth under the condition of H_2_S addition was considered to be attributed to the sulfide in the medium as a sulfur source because H_2_S exposed in the test tubes, regardless of the presence of mycelium, disappears within a few hours due to hydrolysis by water in the medium. In addition to the growth estimated based on the amounts of ergosterol, the colony appearance of strain THIF08 was also affected by the concentration of COS (Fig. S2). Consequently, we conclude that strain THIF08 can utilize gaseous COS as a sulfur source and that its growth generally corresponds to the amount of COS added, but excessive amounts of COS had an adverse effect (Fig. 1a through e).

### COSase-catalyzed fungal sulfur assimilation

**Fig 2:**
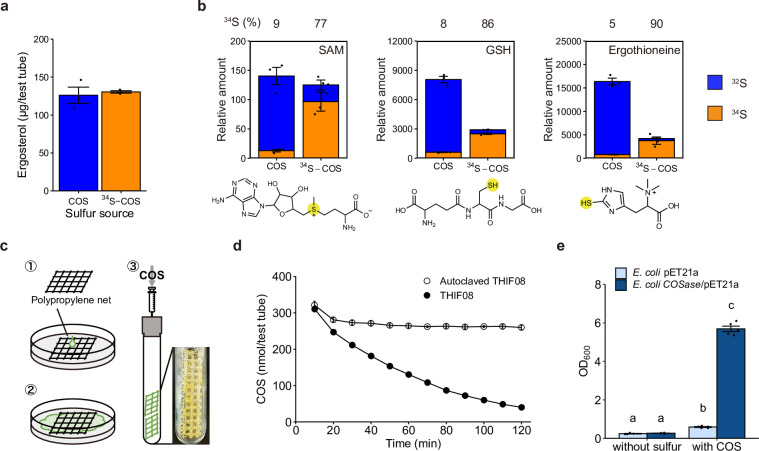
Uptake and assimilation of COS into cells. (a) Comparison of biomass expressed by the amounts of ergosterol between cells cultured with natural abundance COS or ^34^S-COS as a sole sulfur source. Strain THIF08 was cultivated for 4 days with COS or ^34^S-COS (*n* = 3). (b) Comparison of isotopic ratios of ^32^S and ^34^S in sulfur metabolites between cells cultured with natural abundance COS and ^34^S-COS as a sole sulfur source. Strain THIF08 cultivated with COS or ^34^S-COS for 4 days was analyzed by LC-MS/MS after derivatization with mBBr. The relative abundances of ^32^S and ^34^S in three sulfur metabolites, SAM, GSH, and ergothioneine, that were detected in the cells with COS or ^34^S-COS were shown (*n* = 3). (c) Schematic diagram of the experiment in which mycelia were separated from the medium. Strain THIF08 cultured on a polypropylene net over the agar medium was removed from the surface of the medium after the whole area of the polypropylene net was covered with mycelium. (d) COS degradation on a polypropylene net after the removal of the medium. Filled circles, strain THIF08 on a polypropylene net; open circles, autoclaved strain THIF08 on a polypropylene net (*n* = 3). (e) Effects of *COSase* insertion on *E. coli* growth in the culture supplemented with COS as the sole sulfur source. *E. coli COSase*/pET21a and *E. coli* pET21a were cultured with 1.6 µmol of COS or without COS in a 2 L headspace; cells were collected in PBS buffer, and the OD_600_ of the obtained bacterial suspension was measured to evaluate bacterial growth (*n* = 5). Statistical significance was determined using a one-way ANOVA followed by Tukey’s *post hoc* test to identify specific group differences, with a significance level set at *P* < 0.05. In panels a, b, d, and e, data are means ± s.e.m. *n* represents independent biological replicates.

To trace the fate of sulfur atoms after fungal COS uptake, we cultured strain THIF08 with 190 nmol of ^34^S-labeled synthesized COS isotopologue (^34^S > 99.9%, ^34^S-COS thereafter) (33) or natural abundance COS (^32^S, 95.0%; ^33^S, 0.75%; ^34^S, 4.2%; and ^35^S, 0.015%) (34) in 22 mL gas volume. There was no significant difference in ergosterol accumulation between cultivation with natural abundance COS and ^34^S-COS after 4 days incubation, which indicates that ^34^S-COS does not inhibit fungal growth (Fig. 2a). Sulfur metabolomes were analyzed using liquid chromatography tandem mass spectrometry (LC-MS/MS) combined monobromobimane (mBBr), which specifically derivatizes thiol [the sulfur index analysis, thereafter, see Materials and Methods (35)]. The results showed that ^34^S from COS was introduced into sulfur metabolites such as S-adenosylmethionine (SAM), glutathione (GSH), and ergothioneine (Fig. 2b). The production of ^34^S-sulfur metabolites in fungal cells strongly suggests that COS assimilation had occurred.

COS can be abiotically hydrolyzed to H_2_S, hydrosulfide ion, and/or sulfide ion by water in the agar medium (36). To exclude the possibility of indirect transfer of sulfur from COS via the hydrolysates of COS into fungal cells, we inoculated strain THIF08 onto a polypropylene net placed on the agar medium, removed the polypropylene net covered with mycelium after fungal growth (Fig. 2c), transferred the fungal mycelia into a glass test tube (thus separating it from the agar medium), added COS into headspace of the glass tube, and measured changes in COS concentration in the headspace. A decrease in COS was still observed, even though there was no possible COS hydrolysis via contact with water in the medium (Fig. 2d). No degradation of COS was observed in mycelium that was heat inactivated by autoclaving. These results suggest that the fungal COS uptake occurs via a direct pathway into the cell, which further supports the occurrence of gaseous sulfur assimilation.

We found a COSase in strain THIF08 that specifically acts on COS (31) and hypothesized that this enzyme is responsible for the initial reaction of COS assimilation. To confirm the physiological role of COSase in COS assimilation, we created a recombinant *Escherichia coli COSase*/pET21a, using *E. coli* BL21 (DE3) transformed with the plasmid pET21a containing a COSase gene (*COSase*, accession number LC499780 in DDBJ) (31). As a control, we also created *E. coli* pET21a, which was a recombinant *E. coli* BL21(DE3) transformed with the plasmid pET21a that lacks *COSase*. The crude enzyme solution obtained from the transformants showed COSase activity only in *E. coli COSase*/pET21a (Fig. S3). Both transformants were cultivated on agar media with and without the addition of gaseous COS as the sole sulfur source. Although both transformants showed increases in optical density at 600 nm (OD_600_) with COS addition, the growth of *E. coli COSase*/pET21a was approximately 10 times higher than that of *E. coli* pET21a (Fig. 2e). Thus, we conclude that COSase is a key enzyme for fungal COS assimilation and suggest that COS assimilation also occurs in prokaryotes if they produce COSase.

### Energetically efficient sulfur assimilation using gaseous COS

Fungal assimilation of sulfate is an energetically costly process in which sulfate, with an oxidation number of +6, is progressively reduced to sulfide (S^2-^), which has an oxidation number of −2, with the consumption of two ATP and four NADPH (7, 8), and then sulfide is converted into downstream sulfur metabolites. On the other hand, in COS assimilation, a change in oxidation number is not required, because COS already has an oxidation number of −2. Such reductive sulfur assimilation has been studied with thiosulfate and dimethylsulfoniopropionate, whose assimilation is energetically more advantageous than sulfate assimilation because it consumes less ATP and NADPH (37, 38). Thus, if sulfide produced by hydrolysis of COS by COSase is converted to downstream sulfur metabolites, COS is also expected to be a more efficient sulfur source than sulfate.

**Fig 3:**
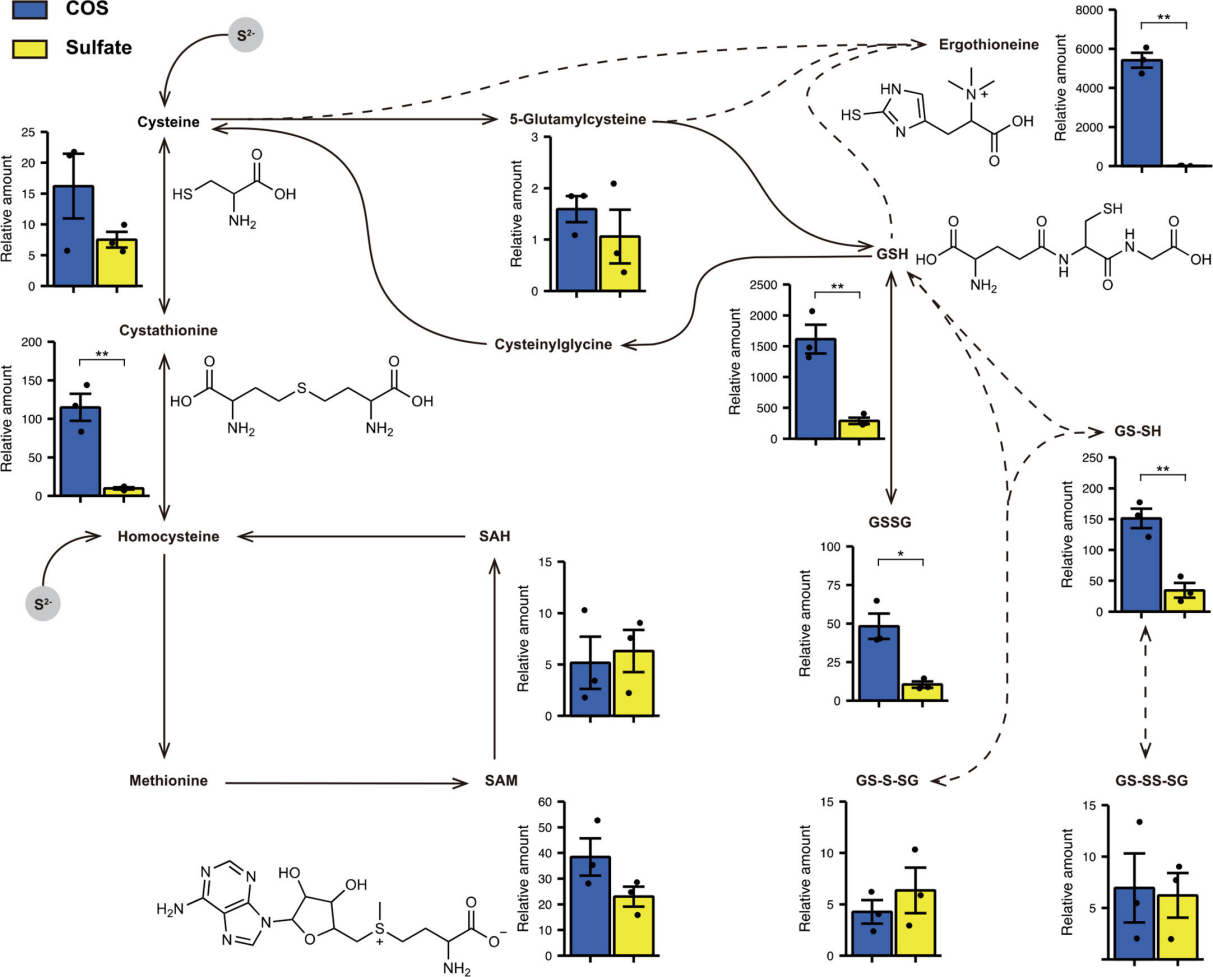
Relative contents of sulfur metabolites estimated by the sulfur index analysis and predicted sulfur metabolic pathway. The contents of thiol metabolites in strain THIF08 cultured with COS or sulfate as a sole sulfur source were expressed as relative amounts by the peak area in the mass spectrometry monitoring the *m*/*z* transition of the derivatives, and the values were normalized to those amounts of ergosterol. Data are means ± s.e.m. of three biological replicates for relative contents of cysteine, cystathionine, S-adenosylmethionine, S-adenosylhomocysteine (SAH), 5-glutamylcysteine, glutathione, glutathione disulfide (GSSG), glutathione persulfide (GS-SH), a persulfide form of glutathione disulfide (GS-S-SH), GS-SS-SG, and ergothioneine are shown. Statistical significance was calculated using a two-tailed Student’s *t*-test if the *F*-test was *P* > 0.05 or a two-tailed Welch’s *t*-test if the *F*-test was *P* < 0.05. *P < 0.05 and **P < 0.01.

To compare the metabolic efficiency between COS and sulfate, the sulfur compounds extracted from strain THIF08, cultured for 7 days in COS-supplemented or sulfate-supplemented conditions as a single sulfur source, were analyzed by the sulfur index analysis (39–41) (Fig. 3). For both cultures, sulfur-containing metabolites such as cysteine and the downstream metabolites of cysteine were detected. No metabolites were more abundant in the sulfate-supplemented culture than in the COS-supplemented culture. On the other hand, the production of GSH, which functions as an antioxidant and a storage substance for sulfur elements (2), and its persulfide were significantly more abundant in the COS-supplemented culture than in the culture supplemented with sulfate. In addition, the production of ergothioneine, which is also a stable antioxidant (42), is about 500 times higher in the COS-supplemented culture than in the sulfate-supplemented culture. The relative abundance of reduced sulfur compounds under the COS-supplemented conditions suggests that the assimilation of sulfur from gaseous COS is energetically more efficient than sulfur assimilation that starts from sulfate.

### Distribution of fungal COSase and its molecular evolution

COS has been suggested to mediate the formation of peptides from amino acids in prebiotic chemistry (43), but the discovery of gaseous COS assimilation led us to reconsider the roles of COS throughout the Earth’s history. In the geological record, the ppm order of atmospheric COS concentration is suggested in the Archean anoxic atmosphere, which offers a solution to the faint young Sun paradox with COS’s global warming effect (44). In the present atmosphere related to anthropogenic activity, anthropogenic COS sources increased after the industrial revolution (45) and are likely to be a major constituent of the missing source of atmospheric COS (46). Given that COS concentrations have changed throughout Earth’s history, the importance of sulfur acquisition from COS could be different across geological time scales.

**Fig 4:**
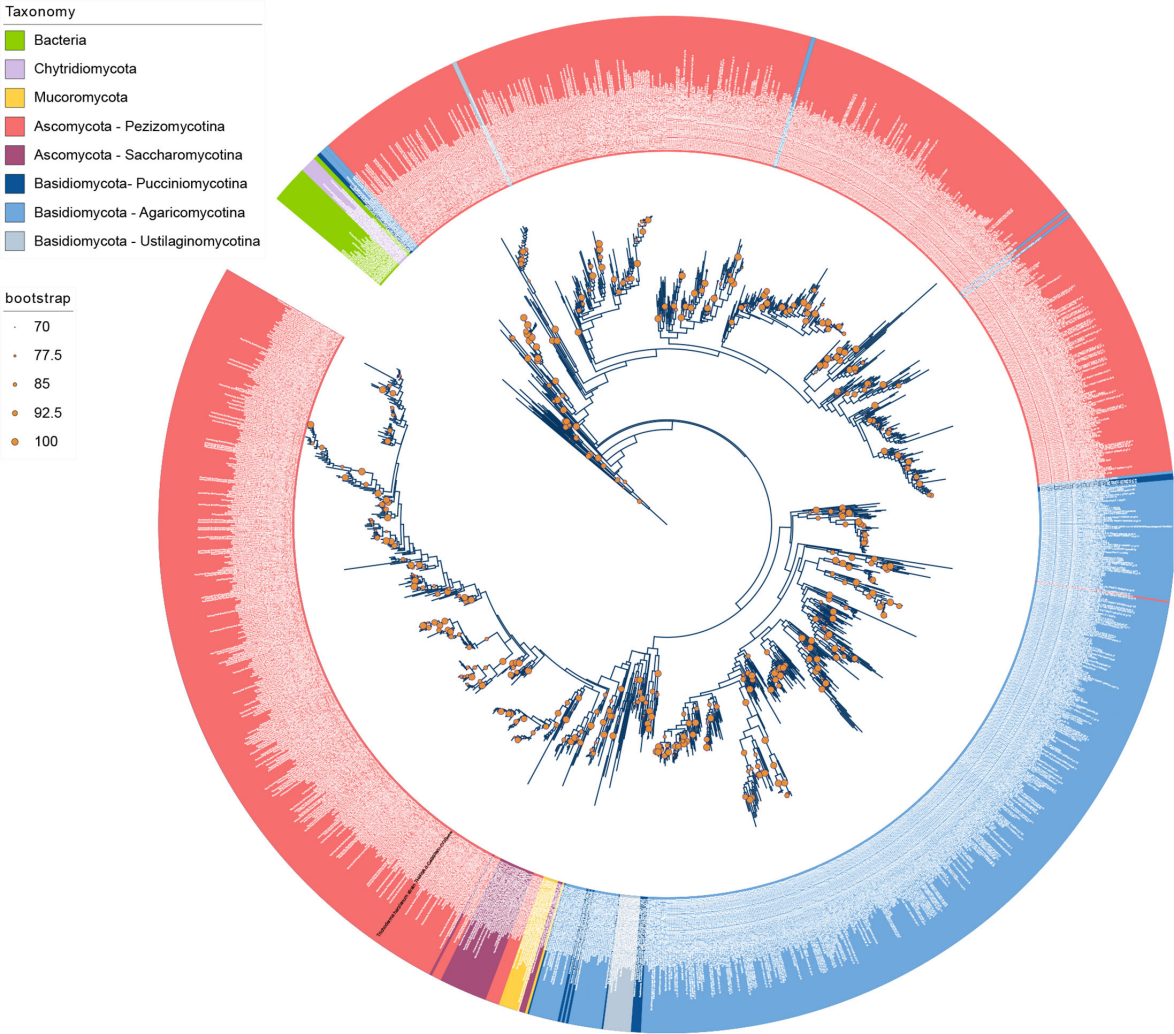
Phylogenetic tree of COSase-like genes, COSase, CS_2_ hydrase, and clade D β-CA family enzymes. Fungal sequences were retrieved from the JGI MycoCosm database via a blastp search, using the amino acid sequence of strain THIF08 COSase as a query. Among the 1,675 fungal sequences retrieved, sequences without a starting methionine or a stop codon and sequences that lacked the Cys 36, His 88, and Cys 91 residues of strain THIF08 (which coordinate with the Zn active center of the enzyme) were trimmed and redundant sequences were removed, yielding a data set of 1,476 sequences [including 20 prokaryotic β-CA family enzyme sequences, corresponding to “clade D” of Masaki et al. (31)], with an aligned length of 175 amino acids. Taxonomic distribution of sequences is indicated by colored backgrounds for labels of terminals, and bootstrap support is indicated by yellow circles on branches (see figure for legend).

To elucidate the evolution of fungal COSase, we performed phylogenetic analyses of fungal COSase sequences. A search of the MycoCosm (47) genome portal retrieved 1,455 sequences encoding COSase-like enzymes that are broadly distributed among three phyla of filamentous fungi (Ascomycota, Basidiomycota, and Mucoromycota) and certain zoosporic Chytridiomycota (Table S1). Although it has been reported that only Ascomycota degrade COS, our assay for COS-degrading activity with several basidiomycetous fungi showed that all the tested fungi degraded COS (Fig. S4). This supports the statement above that COSase is widely distributed in the fungal taxa in terms of its degrading activity. Bootstrap support for many deep nodes in the COSase-like gene phylogeny is weak, which limits inferences about gene family evolution (Fig. 4; Fig. S5). Nevertheless, it is noteworthy that in addition to Basidiomycota, Ascomycota, and Mucoromycota, the anaerobic gut-inhabiting Neocallimastigomycetes, which is a group of the evolutionarily ancient Chytridiomycota, also had COSase-like genes. Prokaryotic sequences formed a paraphyletic grade, which is consistent with the view that fungi acquired COSase-like genes via horizontal gene transfer (HGT) from Bacteria. Neocallimastigomycetes are placed with strong support (bootstrap = 99%) in a clade containing three sequences from prokaryotes including the type I methanotroph *Methylococcus capsulatus* (48), which is associated with herbivore rumens (49, 50), suggesting a possible ecological context for HGT from bacteria to Neocallimastigomycetes fungi, as has been shown for other genes (51). On the other hand, amino acid sequences of COSase-like enzymes from Ascomycota, Basidiomycota, and Mucoromycota form a separate, weakly supported clade, with no obvious closely related prokaryotic sequences. The presence of genes encoding COSase-like enzymes in these three phyla suggests that they were present in the common ancestor of the filamentous fungi (excluding Zoopagomycota). COSase-like sequences were not detected from Glomeromycotina (Mucoromycota), Taphrinomycotina (Ascomycota), and certain other major groups (Table S1), implying that they may have been lost repeatedly during fungal evolution.

Phylogenetic analysis of COSase genes suggested that the common ancestor of Ascomycota, Basidiomycota, and Mucoromycota possessed COSase. Based on recent molecular clock analysis using fungal genome information, the time of divergence of Dikarya (Ascomycota plus Basidiomycota) and Mucoromycota is estimated to have occurred roughly at 790–670 Ma (52). This coincides with the time early fungi transitioned to terrestrial environments (52). During this period, the concentration of oxygen in the atmosphere was estimated to be increased (53). Since COS in the troposphere is stable and long-lived in oxygen-rich atmospheres, it is possible to estimate that COS was relatively uniformly distributed on a global scale in the atmosphere at around 790–670 Ma as it is today, although the exact COS concentrations during that period are unknown. It is hypothesized that fungi underwent evolutionary adaptations, such as the loss of flagella, to suit terrestrial environments (3, 52, 54). An additional adaptation to the terrestrial environment could have involved fungi acquiring the ability to assimilate atmospheric COS, which was uniformly present. We note that prokaryotes carrying COSase genes may also be capable of COS assimilation, and several bacteria (19, 20) have been found to degrade COS on the order of ppm, as well as fungi (23, 24). Thus, prokaryotes may have acquired the ability to degrade COS by adapting to Archaean anoxic atmospheric conditions with higher COS concentrations than today, with fungi following in the Neoproterozoic Era.

### Conclusion

The results of this study demonstrate that the filamentous fungus *T. harzianum* strain THIF08 can grow with gaseous COS as the sole sulfur source and that it can assimilate it for a variety of sulfur-containing metabolites. It was also shown that this process is not a water-mediated process in the medium but a direct uptake of COS in a gaseous form, and that COSase is the key enzyme to the degradation. Fungal COSase was distributed among three phyla of filamentous fungi (Ascomycota, Basidiomycota, and Mucoromycota) and certain zoosporic Chytridiomycota, and phylogenetic analysis of fungal COSase estimated the common ancestor of filamentous fungi, which acquired COSase roughly 790–670 Ma.

While COS is present in the atmosphere at concentrations of around 500 pptv, the experiments in this study were conducted under significantly higher concentration conditions. Although further research on COS metabolism under more natural conditions is needed, this study demonstrates that fungi have the ability to assimilate gaseous sulfur compounds, similar to other assimilation processes, such as carbon fixation and nitrogen fixation. Like N_2_O for N_2_O-reducing microorganisms (55), COS has been known to be used for energy metabolism in chemolithoautotrophic bacteria (18, 22). As a gaseous sulfur molecule, H_2_S is also known to be produced intracellularly and used as a signaling molecule, as are NO and CO (3, 56, 57). However, these are not the processes that incorporate sulfur taken in from the atmosphere into metabolites, and therefore, at least the COS assimilation process we have found in this study is the first gaseous sulfur compound assimilation process. In addition, it was suggested that the COS assimilation pathway is more energy efficient than sulfate assimilation because it does not involve a redox change of sulfur. Although both carbon fixation using CO_2_ and nitrogen fixation using N_2_ involve redox reactions, we have not seen any examples of redox reactions being incorporated as one of the factors in the definition of fixation. Thus, the findings in this study regarding the existence of gaseous COS assimilation processes by fungi, while requiring further study, maybe one piece of evidence for the existence of a fixation pathway for gaseous sulfur molecules, i.e., sulfur fixation.

## MATERIALS AND METHODS

### Fungal strain

*T. harzianum* strain THIF08 was isolated from forest soil in our previous study (24) and was maintained on potato dextrose agar (PDA, Nihon Pharmaceutical, Osaka, Japan). This strain was used to investigate COS degradation, fungal growth, COS uptake, and the COS metabolic pathway.

### Preparation of spore suspension

To prepare the spore suspension, *T. harzianum* strain THIF08 was cultivated on a PDA plate medium at 25°C in the dark for 10–12 days. After green spores were formed on the surface of mycelia, 2 mL of PBS (1,370 mM NaCl, 26.8 mM KCl, 81 mM Na_2_HPO_4_, and 14.7 mM KH_2_PO_4_, pH 7.4) (NIPPON GENE, Tokyo, Japan) was added to the plate, shaking gently, and 1 mL of the suspension was collected. After centrifugation at 12,000 × *g* for 5 min at 4°C, the supernatant was discarded, and then 1 mL of PBS was newly added followed by mixing with vortex for 10 seconds. The series of sequential operations from centrifugation to suspension was repeated three times to remove the medium-derived components. The number of spores was calculated on a cell counter plate (Thoma type, Watson, Tokyo, Japan), and the spore suspension of 600 cells/µL was used for inoculation of this fungus.

### Cultivation of *T. harzianum* strain THIF08 on an agar medium in a gas-sampling bag

For the cultivation using sampling bags, 20 mL Czapek-Dox agar medium without sulfur [CD(−S); 2.5 g/L NaNO_3_, 1 g/L K_2_HPO_4_, 0.4 g/L MgCl_2_·6H_2_O, 0.5 g/L KCl, 0.01 g/L FeCl_2_·4H_2_O, 15 g/L glucose, and 15 g/L UltraPure Agarose (Invitrogen, Waltham, MA, USA), pH 6.0 adjusted by HCl] was prepared in a Petri dish (approximately 9 cm in diameter), and then 10 µL of the spore suspension prepared as described above was spotted at three locations on the agar medium. The dish was placed in a 5 L Tedlar Bag (GL Sciences) as a sampling bag, which was beforehand cut out on one side and then sterilized with 77% alcohol and UV light. After clipping the cut out, the air in the bag was removed from the collection port of the bag with an aspirator, and then 2 L of air in the clean bench was pumped in with a flex pump (OMI ODOR AIR SERVICE, Shiga, Japan). A butyl rubber plug was attached to the collection port, and COS (9.71%, with N_2_ as the balanced gas, Japan Fine Products, Kanagawa, Japan) was added to make the final COS concentration of 16,000 nmol/bag. The agar medium in the bag was incubated at 25°C in the dark for 2 weeks, and then the amount of COS in the bags and ergosterol extracted from the mycelia were measured, respectively.

### Cultivation of *T. harzianum* strain THIF08 on an agar medium in test tube

For the cultivation using test tubes, 10 mL CD(−S) agar medium was prepared in a 32 mL test tube to be slanted culture. For comparison, different amounts of MgSO_4_·7H_2_O (2, 20, 200, and 2,000 nmol) were added into the media to make a sulfate-containing media [CD(+S)], respectively. Fifty microliters of spore suspension was inoculated to the slanted CD(−S) or CD(+S) medium, and then a butyl rubber plug was attached to each test tube. For CD(−S) agar medium, 2, 20, 200, and 2,000 nmol of COS were added to the 22 mL headspace of the tube. These media were incubated at 25°C in the dark for 8 days, and the ergosterol was extracted from mycelia and the entire agar medium and measured as described below.

### Cultivation of *T. harzianum* strain THIF08 on a polypropylene mesh sheet

To minimize the effects of the medium-derived components including water on COS-degrading activity, strain THIF08 was grown on a polypropylene mesh sheet (5 × 5 cm) that was placed on the surface of the CD(+S) agar medium (0.5 g/L MgSO_4_·7H_2_O was used instead of 0.4 g/L MgCl_2_·6H_2_O). After the entire sheet was covered with fungal mycelia, the sheet together with mycelia was carefully removed from the surface of the medium, and then placed in a 32 mL test tube, which was sterilized by autoclaving beforehand. The test tube was plugged with a butyl stopper and then 260 nmol of COS was added to the tube. For comparison, the mycelia that were heat-inactivated by autoclaving (121°C, 30min) were also manipulated in the same manner. The amount of COS in the headspace was measured by gas chromatography equipped with a flame photometric detector (GC-FPD) every 10 min during the first 2 h of COS addition.

### Determination of the amount of COS in the headspace of cultures

The amount of COS in the headspace of cultures was measured by GC-FPD (GC-14B, Shimadzu, Kyoto, Japan). A gas sample in the headspace was collected using a gas-tight syringe (Ito Corporation, Shizuoka, Japan) fitted with a needle (XX-MS61, Ito Corporation). The contents of the syringe were injected into a gas chromatograph composed of the column (length, 2 m; inner diameter, 3.0 mm) packed with Sunpak-S (Shinwa Chemical Industries, Kyoto, Japan). The gaseous compounds were separated using N_2_ gas (>99.999%, Ichimura Sanso Co., Ltd, Tokyo, Japan) as a carrier gas at the flow pressure of 260 kPa at the column temperature of 70°C, and then the amount of COS was quantified by comparing it to the square root of the peak height of standards containing 200 ppmv.

### Determination of the amount of ergosterol extracted from mycelia

To monitor the growth of strain THIF08, ergosterol, which is the specific membrane sterol of the fungal cell, was measured. Ergosterol was extracted from the collected mycelia according to the procedure of Montgomery et al. (58). The mycelia were collected from the entire area of the medium, added to 1.4 M KOH in methanol-ethanol (5:3, vol/vol), mixed, and placed in a water bath at 75°C for 1 h. After immersing in water and cooling to room temperature, ergosterol was extracted in a total of 15mL of *n*-pentane, evaporated, and then dissolved into 1mL of methanol-tetrahydrofuran (1:1, vol/vol) before high performance liquid chromatography (HPLC) analysis (LC-10A, Shimadzu). Ergosterol was separated on a Zorbax ODS column (250 mm × i.d. 4.6 mm) with methanol (>99.7%, FUJIFILM Wako Pure Chemical Corp., Osaka, Japan) as the mobile phase at a flow rate of 1 mL min^−1^ and was monitored at 282 nm. Ergosterol [>98.0%, (for HPLC), FUJIFILM Wako Pure Chemical Corp.] was used as a standard (*R*^2^ > 0.9999).

### Sulfur index analysis of metabolites from *T. harzianum* strain THIF08

To investigate the assimilation of COS-derived S, sulfur metabolites in mycelia grown under conditions of ^34^S-labeled synthesized COS isotopologue or natural abundance COS were examined. The LC-MS/MS analysis of sulfur metabolites was prepared by thiol-specific S-bimanyl derivatization with mBBr, as per sulfur index analysis (35) as follows.

Strain THIF08 was inoculated with 50 µL of spore suspension in slanted CD(−S) agar medium and then incubated at 25°C in the dark. After 3 days of incubation, the silicone stopper was replaced with a butyl stopper, and 190 nmol of natural abundance COS or ^34^S-COS, which was ^34^S-labeled COS created carbon monoxide with elemental ^34^S (^34^S > 99.9%) according to the method of Hattori et al. (33), was added respectively. After 4 days of incubation, the mycelium on the surface of the medium was peeled off with a microspatula, 3 mL of methanol was added to suspend the mycelia, and the mycelia were then collected. The cell suspension was sonicated on ice for 1 min, and sulfur index analysis was carried out by using 0.5 mL of the suspension. The suspension was centrifuged (16,100 × *g*) at 4°C for 3 min, and 100 µL of the supernatant containing extracted metabolites was subjected to the reaction with 10 µL of 20 mM mBBr, 10 µL of 0.2 M Tris, and 10 µL of MQ. The resulting solution was vortexed for 30 seconds and inverted for 10 min. After drying, the solidified metabolites were resuspended with 50 µL of MQ water, and then 5 µL was subjected to LC-MS/MS analysis (LC-MSMS 8030, Shimadzu). The relative amount of the target metabolite was determined from the peak area of mass chromatogram monitoring the respective *m*/*z* transitions and normalized by the amounts of ergosterol. The *m*/*z* of ^34^S-containing sulfur metabolites (glutathione, ergothioneine, and S-adenosylmethionine) were detected by adding two to the usual *m*/*z* ^32^S-containing ones. The rest of the methanol suspension was used for ergosterol quantification as described above.

To compare sulfur metabolites in the mycelia of strain THIF08, grown either with COS or sulfate as a sole sulfur source, we conducted a sulfur index analysis. We inoculated 50 µL of the spore suspension of strain THIF08 into a slanted CD(−S) medium in a test tube with a silicone stopper and incubated it at 25°C in the dark. After incubation for 3 days, the silicone stopper was replaced with a butyl stopper and 190 nmol of COS was added. For comparison, we used a slanted CD(+S) medium in the same way but without the addition of COS. One week after inoculation, mycelium was collected and subjected to the sulfur index analysis.

### Preparation of recombinant *E. coli* introduced with COSase gene

Total RNA was extracted from strain THIF08 cultivated in a PDA medium at 25°C for 8 days by using the RNeasy plant mini kit (Qiagen, Hilden, Germany). First-strand cDNA was synthesized using reverse transcriptase (SuperScriptIV; Invitrogen) with oligo(dT)_20_ primer (Invitrogen). The following two oligonucleotide primers were designed, based on the nucleotide sequence of the COSase gene from strain THIF08 (31), for subcloning into the *E. coli* expression vector pET21a: primer 5′ (5′-AAGGAGATATACATATGACCGTCGCCAGCGAG-3′) and primer 3′ (5′-TGCTCGAGTGCGGCCGCTTAAACATCAACCTTGTTAATCTTGCCCG-3′). The target fragments amplified by PCR using KOD-Plus-Neo (TOYOBO, Osaka, Japan) were inserted into the *Nde*I and *Not*I sites of pET21a using the In-Fusion HD cloning kit (TaKaRa Bio, Shiga, Japan). The resultant plasmid (*COSase*/pET21a) was used for the transformation of *E. coli* BL21(DE3) (New England Biolabs, Beverly, MA, USA), and the obtained transformant was designated *E. coli COSase*/pET21a. For comparison, pET21a was also introduced into *E. coli* BL21(DE3), and the obtained transformant was designated *E. coli* pET21a. These transformants were used to investigate the effects of *COSase* insertion on *E. coli* growth.

### Cultivation of recombinant *E. coli* introduced with COSase gene under COS

*E. coli COSase*/pET21a and *E. coli* pET21a were grown in 10 mL of LB medium containing ampicillin (0.1 mg/mL) for 18 h at 37°C with shaking. After centrifugation at 1,000 × *g* for 5 min at 4°C, the supernatant was discarded, and 5 mL of PBS was newly added and gently mixed by inverting. The series of operations from centrifugation to suspension was repeated five times to remove the medium-derived components. The OD_600_ values of the cell suspensions were measured using a spectrophotometer U-3900 (Hitachi, Tokyo, Japan) to confirm that there is no difference in bacterial cell density between recombinants. Both cell suspensions were diluted 100-fold in PBS buffer containing 3 mM IPTG. A volume of 0.5 mL of the prepared cell suspension was inoculated to sulfur-free M9 synthetic agar medium (17.2 g/L Na_2_HPO_4_·12H_2_O, 3.0 g/L KH_2_PO_4_, 1.0 g/L NH_4_Cl, 0.5 g/L NaCl, 0.2 g/L MgCl_2_·6H_2_O, 0.011 g/L CaCl_2_, 4 g/L glucose, 15 g/L UltraPure Agarose). The petri dish was placed in a Smart PA Bag as a sampling bag, and then the gas phase was conditioned to 2 L, as described above, followed by the addition of 1,600 nmol COS into the gas phase (final concentration is about 20 ppmv). As a comparison, a culture condition without COS addition was also prepared. *E. coli COSase*/pET21a in the bag was stationarily cultured at 37°C in the dark. After 24 h incubation, 3 mL of PBS buffer was added to the cultured petri dish, and the cells were suspended by pipetting and then collected. The cell collection process was repeated with different amounts of PBS buffer added (3, 2, and 1 mL), and finally, 4 mL of cell suspension was obtained. *E. coli* pET21a was also cultured well as *E. coli COSase*/pET21a. The OD_600_ of the suspensions was compared between *E. coli COSase*/pET21a and *E. coli* pET21a.

### Phylogenetic analyses

Fungal sequences were retrieved from the JGI MycoCosm (47) database via a blastp search with the expected value set to 10^−25^, using the amino acid sequence of strain THIF08 COSase as a query. Sequences without a starting methionine or a stop codon were excluded, as were sequences that lacked the Cys 36, His 88, and Cys 91 residues of strain THIF08 (which coordinate with the Zn active center of the enzyme). Fungal sequences thus retrieved were combined with 20 prokaryotic β-CA family enzyme sequences, corresponding to “clade D” of Masaki et al. (31), and aligned using MAFFT version 7 (59) with the L-INS-I algorithm. Aligned sequences were submitted to ModelTest NG (60) on CIPRES (61) and trimmed using Trimal v.1.36 on Phylemon2 (62). Phylogenetic analyses were conducted using RAxML version 8.2.12 (63) on CIPRES5, with the LG substitution matrix, gamma rate heterogeneity, 1,000 rapid bootstrap replicates, and maximum likelihood optimization. Trees were rooted with eight bacterial sequences, based on the results of Masaki et al. (31). Phylogenetic trees were visualized, and figures were created using iTOL v6 (64).

### Statistical analysis

Results are presented as means ± s.e.m. For statistical significance comparisons between two groups, we used a two-tailed Student’s *t*-test if the *F*-test was *P* > 0.05or a two-tailed Welch’s *t*-test if the *F*-test was *P* < 0.05, with significance set at *P* < 0.05. For comparisons involving multiple groups, a one-way ANOVA was conducted followed by Tukey’s *post hoc* test to determine specific group differences.

## Data Availability

No new code was used in this study.

## SUPPLEMENTAL MATERIAL

Fig. S1 to S4 and Table S1 aem.02015-23-s0001.docx Fig. S1 to S4 and Table S1.

Fig. S5 aem.02015-23-s0002.pdf Figure S5.

ASM does not own the copyrights to Supplemental Material that may be linked to, or accessed through, an article. The authors have granted ASM a non-exclusive, world-wide license to publish the Supplemental Material files. Please contact the corresponding author directly for reuse.
