# Supplementary material for: Sulfur assimilation using gaseous carbonyl sulfide by the soil fungus Trichoderma harzianum
Source: Appl Environ Microbiol. 2024 Feb 1;90(2):e02015-23. doi: 10.1128/aem.02015-23 (PMC10880591; doi:10.1128/aem.02015-23)
Supplement: Fig. S1 to S4 and Table S1 [file aem.02015-23-s0001.docx]

**Supplementary Methods**

**For Fig. S1**

For cultivation of strain THIF08 supplemented with COS or H_2_S, 50 μL spore suspension (600 spores/μL) was inoculated to 10 mL CD(-S) slanted medium in a 32 mL-volume test tube. A butyl rubber plug was attached to the test tube, and then 200 nmol of gaseous COS or H_2_S were added to the 22 mL headspace. Those test tubes were incubated at 25°C in the dark for 8 days, and ergosterol was extracted and measured by HPLC as described in Methods.

**For Fig. S2**

To examine the dose effects of COS concentration on the colony appearance of strain THIF08, 10 μL of spore suspension (600 spores/μL) was spotted at three locations on a 20 mL CD(-S) agar medium in a Petri dish. The dish was placed into a 5 L-Smart Bag PA (GL Sciences). The air in the bag was drawn out with an aspirator, and then 2 L of air in the clean bench was pumped in with a flex pump. A butyl rubber plug was attached to the collection port of the bag, and 0.16, 16, 160, 1,600, 16,000, and 160,000 nmol COS was added, respectively. The agar media in the bag were incubated at 25 °C in the dark for 14 days. As a comparison, CD(+S) medium containing 16,000 nmol sulfate in the agar medium in a Petri dish was prepared.

**For Fig. S3**

*E. coli* *COSase*/pET21a and *E. coli* pET21a were grown to the mid-exponential phase in 100 mL of LB medium containing ampicillin (0.1 mg/mL) and IPTG was added to the culture at a final concentration of 0.1 mM to induce the production of COSase. Cells were harvested by centrifugation at 10,000 ×g at 4°C for 5 min and re-suspended in 5 mL of 20 mM Tris-HCl buffer, pH 8.0. The cells were disrupted by sonication, centrifuged at 10,000 ×g at 4°C for 5 min, and the supernatant was used as the crude extract. The protein content of the crude extracts was measured using a Pierce™ BCA Protein Assay Kit (Thermo Fisher Scientific). COSase activity was measured by GC-FPD as described in Methods.

**For Fig. S4**

For the assay of COS degrading activity, 9 species of basidiomycetes (*Gloeophyllum trabeum* NBRC 6430, *Coniophora puteana* MAFF 420262, *Fomitopsis palustris* FFPRI T0507, *Fomitopsis pinicola* NBRC 106830, *Neolentinus suffrutescens* NBRC 110193, *Wolfiporia cocos* NBRC 30268, *Serpula lacrymans* MAFF 420003, *Trametes versicolor* NBRC 30340, and *Pleurotus ostreatus* NBRC 30776), and one ascomycete *Trichoderma harzianum* strain THIF08 were inoculated on 10 mL PDA slanted medium in a test tube, and incubated for 1-2 weeks until mycelium covered the entire medium. After a silicone stopper was replaced with a butyl stopper, 180 nmol COS was added. One hour after the addition of COS, the amount of COS and H_2_S in the headspace of each test tube was measured by GC-FPD as described in Methods.

**Supplementary Data**

**Fig. S1 Growth of strain THIF08 using COS or H_2_S as the sole sulfur source.** Strain THIF08 was cultivated for 8 days on 10 ml CD(-S) slanted medium supplemented with 200 nmol COS or H_2_S in 22 mL headspace of test tubes (final concentration 220 ppmv) as the sole sulfur source, respectively. Data are means ± s.e.m. of four biological replicates. Statistical significance was calculated using a two-tailed Student's t-test. **P* < 0.01.

**Fig. S2 Colony appearance of strain THIF08 at various concentrations of COS.** Strain THIF08 was cultivated on 10 mL CD(-S) agar medium with various amounts of COS in the 2 L headspace of Smart PA bag for 14 days. In conditions with sulfate as a single sulfur source, CD(+S) agar medium containing sulfate was placed in 2 L Smart PA bags without the addition of COS.

**Fig. S3 Time course of COS degradation and H_2_S production by crude extract of recombinant *E. coli*.** Sampling of the headspace gas was started 2 min after the addition of COS. The amount of protein used for the assay was 0.30 μg for *E. coli COSase*/pET21a (filled circles) and 0.23 μg for *E. coli* pET21a (open circles). Tris-HCl buffer, pH 8.0 as negative control (open square). COS (solid line); H_2_S (dotted line). Data are means ± s.e.m. of three replicates.

**Fig. S4 COS degradation by various fungi.** Ten fungal species were cultivated on PDA slant medium. After mycelium covered the entire surface of the medium, 180 nmol COS was added to the headspace and amounts of COS and H_2_S were measured. Figure indicates the residual COS after 1 hour incubation. Control indicates uninoculated condition. ND means not detection. Statistical significance was determined using a one-way ANOVA followed by Tukey's post-hoc test to identify specific group differences, with a significance level set at *P* < 0.05. *T. versicolor* NBRC 30340, *P. ostreatus* NBRC 30776, and *S. lacrymans* MAFF 420003 released H_2_S transiently. *S. lacrymans* MAFF 420003 degraded 180 nmol COS within 40 min.

**Table S1 Numbers of COSase-like genes in fungal genomes.** Homology searches were performed via Blastp with the Expect value set to 10^-25^ against the amino acid sequence of each Class/Subdivision from the JGI MycoCosm database, using COSase from *T. harzianum* strain THIF08 as a query. Sequences without a starting methionine or a stop codon were excluded, as were sequences that lacked the Cys 36, His 88, and Cys 91 residues of *T. harzianum* strain THIF08 (which coordinate with the Zn active center of the enzyme).

**(Attached separately)**

**Fig. S5 Phylogenetic tree of COSase-like genes, COSase, CS_2_ hydrase, and clade D β-CA family enzymes in rectangular form.** This has the same topology as the circular phylogenetic tree in Fig. 4.
