# Supplementary material for: Sulfur assimilation using gaseous carbonyl sulfide by the soil fungus Trichoderma harzianum
Source: Appl Environ Microbiol. 2024 Feb 1;90(2):e02015-23. doi: 10.1128/aem.02015-23 (PMC10880591; doi:10.1128/aem.02015-23)
Supplement: Fig. S5 — Figure S5. [file aem.02015-23-s0002.pdf]

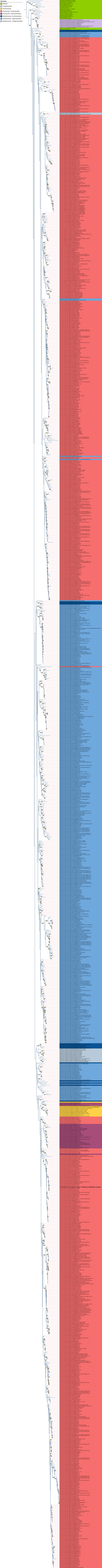
Taxonomy
Bacteria
Chytridiomycota
Mucoromycota
Ascomycota - Pezizomycotina
Ascomycota - Saccharomycotina
Basidiomycota- Pucciniomycotina
Basidiomycota - Agaricomycotina
Basidiomycota - Ustilaginomycotina
